# Increased Brain Neurotensin and NTSR2 Lead to Weak Nociception in NTSR3/Sortilin Knockout Mice

**DOI:** 10.3389/fnins.2016.00542

**Published:** 2016-11-24

**Authors:** Christelle Devader, Sébastien Moreno, Morgane Roulot, Emmanuel Deval, Thomas Dix, Carlos R. Morales, Jean Mazella

**Affiliations:** ^1^CNRS, Institut de Pharmacologie Moléculaire et Cellulaire, UMR 7275, Université de Nice Sophia AntipolisValbonne, France; ^2^Department of Drug Discovery and Biomedical Sciences, College of Pharmacy, Medical University of South CarolinaCharleston, SC, USA; ^3^JT Pharmaceuticals, Inc.Mount Pleasant, SC, USA; ^4^Department of Anatomy and Cell Biology, McGill UniversityMontreal, QC, Canada

**Keywords:** neurotensin, receptor, sortilin, knockout gene, nociception

## Abstract

The neuropeptide neurotensin (NT) elicits numerous pharmacological effects through three different receptors (NTSR1, NTSR2, and NTSR3 also called sortilin). Pharmacological approaches and generation of NTSR1 and NTSR2-deficient mice allowed to determine the NT-induced antipsychotic like behavior, the inhibitory of weak fear memory and the nociceptive signaling in a rat formalin tonic pain model to NTSR1. Conversely, the effects of NT on thermal and tonic nociceptions were mediated by NTSR2. However, the role of NTSR3/sortilin on the neurotensinergic system was not investigated. Here, by using C57Bl/6J mouse model in which the gene coding for NTSR3/sortilin has been inactivated, we observed a modification of the expression of both NTSR2 and NT itself. Quantitative PCR and protein expression using Western blot analyses and AlphaLisa™ technology resulted in the observation that brain NTSR2 as well as brain and blood NT were 2-fold increased in KO mice leading to a resistance of these mice to thermal and chemical pain. These data confirm that NTSR3/sortilin interacts with other NT receptors (i.e., NTSR2) and that its deletion modifies also the affinity of this receptor to NT.

## Introduction

The endogenous neuropeptide NT is involved in numerous biological functions both in the brain and in periphery organs (for review see Kleczkowska and Lipkowski, 2013). These processes include dopamine transmission (Kitabgi et al., 1989), analgesia (Dobner, 2006), hypothermia (Popp et al., 2007) and hormonal activity regulation (Rostene and Alexander, 1997; Beraud-Dufour et al., 2010). The effects of NT are the consequence of its interaction with three different NT receptors (NTSRs). NTSR1 and NTSR2 are both seven transmembrane (TM) domain G protein-coupled receptors (GPCR) whereas NTSR3 is a single TM domain type I receptor that displays 100% homology with the sorting protein, sortilin (Petersen et al., 1997; Mazella et al., 1998; Mazella, 2001).

The use of selective agonists and antagonists as well as the generation of NTSR1 and NTSR2-deficient mice permitted the determination of the role of these two GPCRs in the NT-induced central effects. The high affinity NTSR1, insensitive to levocabastine, is involved in a series of actions of NT including the antipsychotic like behavior (Mechanic et al., 2009), the inhibition of weak fear memory (Yamada et al., 2010) and the nociceptive signaling in a rat formalin tonic pain model (Roussy et al., 2008). The deletion in mice of the low affinity NTSR2, sensitive to levocabastine, results in the loss of thermal (Maeno et al., 2004) and tonic nociception of NT (Roussy et al., 2009). Levocabastine is a well characterized compound able to selectively bind by competition with NT to the low affinity NT receptor (i.e., NTSR2) without affecting the binding of NT to NTSR1 in murine brain (Kitabgi et al., 1987; Mazella et al., 1998).

At the level of the neurotensinergic system, NTSR3/sortilin has been shown to interact with NTSR1 to modulate NT signaling in HT29 cells (Martin et al., 2002) and with NTSR2 to contribute to the protective effect of NT in pancreatic beta cells (Beraud-Dufour et al., 2009). NTSR3/sortilin is a protein that belongs to the Vps10p protein family (Marcusson et al., 1994) and displays multiple functions and may act as a receptor or a co-receptor as well as a sorting partner to trigger proteins either to the degradation pathway or to the plasma membrane (reviewed in Mazella, 2001; Hermey, 2009; Carlo et al., 2014; Wilson et al., 2014). Two different NTSR3/sortilin deficient mice have been generated (Nykjaer et al., 2004; Zeng et al., 2009). These mice have been mainly used to study the sorting functions of NTSR3/sortilin including rapid endocytosis of progranulin to lysosomes (Hu et al., 2010; Tall and Ai, 2011).

However, nothing is known about the consequence of NTSR3/sortilin deletion on the neurotensinergic system in mice. Therefore, we investigated the fate of NTSR1, NTSR2 and NT expression in the NTSR3/sortilin-deficient mice developed by the Morales's group (Zeng et al., 2009; Musunuru et al., 2010). In the present study, we observed that the lack of NTSR3/sortilin led to the increase of NTSR2 and NT expression in the adult mouse brain. The higher levels of both NTSR2 and NT in the brain of NTSR3/sortilin-deficient mice resulted, as expected, in the loss of sensitivity of pain measured with thermal and chemical nociceptive tests.

## Materials and methods

### Materials

Neurotensin (NT) was purchased from Peninsula Laboratories. ^125^I-Tyr_3_-NT was prepared and purified as described (Sadoul et al., 1984). The brain permeant JT212 (formerly called ABS212) was kindly provided by Dr. Thomas Dix (Charleston, USA). Levocabastine was generously provided by A. Schotte (Belgium). Bovine Serum Albumin (BSA), mammalian protease and phosphatase inhibitor cocktails were from Sigma France. Rabbit polyclonal antibodies against NTSR1 and NTSR2 were from SantaCruz technologies (USA). The monoclonal antibody against NTSR3 was from BD Bioscience. HRP conjugated goat anti-rabbit and anti-mouse were from Cell Signaling. Sortilin (sort1; Uniprot number: Q6PHU5) knockout mice were kindly provided by Dr. Carlos Morales (Montreal, Canada).

### Binding experiments

Binding experiments were carried out on brain homogenates prepared as previously described (Zsurger et al., 1994). ^125^I-NT (2000 Ci/mmol) has been prepared and purified as described (Sadoul et al., 1984). Homogenates (60 μg of protein) were incubated in 250 μl of 50 mM Tris-HCl, pH 7.5, containing 0.2% BSA and 1 mM MgCl2 at 25°C for 30 min with increasing concentrations of ^125^I-NT alone (from 50 to 400 pM) or isotopically diluted by unlabeled NT (from 0.1 to 25 nM) in the absence or in the presence of levocabastine (1 μM). Binding experiments were terminated by addition of 2 ml ice-cold buffer. Radioactivity bound to homogenate was separated from free ligand by filtration under reduced pressure through cellulose acetate Sartorius filters (SM11107, 0.2 μm pore size). Filters and tubes were rapidly washed twice with 2 ml of incubation buffer. Radioactivity retained on filters was counted with a Packard g-counter. Binding parameters (dissociation constant Kd and maximal binding capacities Bmax) were determined by computerized Scatchard analysis.

### Primer design and real-time qPCR

Mice were killed by cervical dislocation. The brain was dissected and immediately frozen in liquid nitrogen. Total RNA was extracted following the Tri Reagent method (Sigma). 2 μg of total RNA was digested with Turbo Dnase (Ambion) and used as template in the reverse transcription reaction with the SuperScript III Reverse Transcriptase and Random Primers (Invitrogen). Primers (Eurogentec) were specific for sequences of NT, NTSR1 (Uniprot number: O88319), NTSR2 (Uniprot number: P70310), GAPDH and CycloD (Table 1).

**Table 1:** **Oligonucleotides used for qPCR**.

mGAPDH-qPCR-F : AAGAGGGATGCTGCCCTTA
mGAPDH-qPCR-R : TTTTGTCTACGGGACGAGGA
mCycloD-qPCR-F : AAGGATGGCAAGGATTGAAA
mCycloD-qPCR-R : GCAATTCTGCCTGGATAGCTT
mNTs-qPCR-F : TGACTCTCCTGGCTTTCAGC
mNTs-qPCR-R : TCCAGGGCTCTCACATCTTC
mNTR1-qPCR-F2 : GGCAATTCCTCAGAATCCATCC
mNTR1-qPCR-R2 : ATACAGCGGTCACCAGCAC
mNTR2-qPCR-F : TGCACGGTGCTAGTAAGTCG
mNTR2-qPCR-R : AAGGAGACCAGCACGTTCAC

Real-time qPCR was performed on the LightCycler™ 480 (Roche) using the LightCycler™ 480 SYBR Green 1 Master mix (Roche). PCR reactions were performed in 20 μl volume containing 16 ng cDNA, 10 μl 2x LightCycler™ 480 SYBR Green 1 Master mix and 1 μl of primer mix (10 μM forward primer, 10 μM reverse primer). The PCR profile was as follows: 5 min at 95°C, followed by 45 cycles of 10 s at 95°C, 10 s at 60°C and 10 s at 72°C.

The Ct value of each gene of interest was normalized to the Ct of the reference genes as follows: DC = Ct_goi_-Ct_ref_ with Ct_ref_ = (Ct_GAPDH_ x Ct_CycloD_)^(1/2)^ with _goi_ = gene of interest, and _ref_ = reference gene. DDCT = DCT experimental condition - DCT control condition. Values were expressed as 2^−DDCt^ normalized using C57Bl/6J as a control.

### Animals

Adult male mice, weighing 20–25 g (8–10 weeks old) were used in this study. The animals were housed under controlled laboratory conditions (in accordance with the FELASA guidelines and recommendations), 6 mice/cage with a 12 h dark-light cycle, a temperature of 21 ± 2°C, and a humidity of 40–60%. Mice had free access to standard rodent diet and tap water. The NTSR3/sortilin homozygous KO mice were generated by the Morales's laboratory by incorporation of a GFP cassette after exon 1 (Zeng et al., 2009) and the controls were C57Bl/6J male mice from Janvier Labs (St Berthevin, France). All animal care and experimental procedures complied with the policies on the care and use of laboratory animals of European Community legislation 2010/63/EU and were approved by the local Ethics Committee (CIEPAL) (protocol number 00893.02).

### Pain behavioral tests

The writhing test was performed as follows: 20 min prior to acetic acid injection, mice were injected intraperitoneally with either 100 μl of saline or 100 μl of a solution containing 1 μM of JT212, a NT analog able to cross the blood-brain-barrier (Hughes et al., 2010). Writhes were counted over a 15 min period starting from the fifth min after intraperitoneal injection of a 0.5% acetic acid solution (10 μl/g).

The Hot plate test was performed with a hot plate apparatus (Ugo Basile) at 55°C. We measured the time (in seconds) to paw licking and jumping latency in response to heat.

### Determination of blood and central NT concentration

Serum samples were collected in the morning by retroorbital puncture in mice anesthetized by isoflurane 4%. Brain NT was recovered after acid extraction of brain homogenates as described (Kokko et al., 2005). The amount of NT was measured from serum and brain using a method adapted to AlphaScreen technology (Perkin Elmer, France). The technique necessitated the preparation of a biotinylated NT on one hand, and of an antibody against the C-terminus of NT on the other hand.

Rabbit polyclonal antibodies against the C-terminus of NT (2-13) were prepared by Agro Bio (La Ferté St Aubin, France). NT (2-13) (5.4 mg, 3.6 mmol) was solubilized in 1.5 ml of 25 mM phosphate buffer, pH 6.7. N-hydroxysuccinimide biotin (13.5 mmol) resuspended in 700 μl of 70% acetonitrile, 30% dimethyl formamide was added to the peptide solution and incubated overnight at room temperature. Biotin-NT (2-13) was purified by HPLC using a Waters apparatus equipped with a semi-preparative RP18 Lichrosorb column. Biotin-NT (2-13) (eluted at 35 min), identified by mass spectrometry, was collected, quantified by its absorption at 280 nm and lyophylised in aliquots.

According to the principles of AlphaScreen technology, streptavidin-donor microbeads were recognized by biotin-NT (2-13) and the anti-rabbit IgG-acceptor microbeads were bound by anti-NT (2-13) antibodies. The signal was produced when the two microbeads (acceptor and donor) were drawn into proximity by a molecular interaction occurring between the binding partners captured on the beads. The peptide present in the sample was able to interfere with this interaction leading to competition. Standard curves were obtained by incubation in 96-well plaque of 1 nM biotin-NT (2-13) with the anti-NT (2-13) antibody (1:5000) in the AlphaLisa™ buffer in the absence or in the presence of increasing concentrations of NT (2-13) (from 10^−11^ to 10^−6^ M) for 1 h at room temperature. After addition of acceptor and donor beads and further incubation for 2 h at room temperature, the plaque was read using the Enspire apparatus (Perkin). Note that non-apparented peptides like somatostatin or spadin were unable to interfere with the dosing method. For sera measurements, the same volume of serum was added instead of unlabeled NT (2-13). The amount of NT was determined from its percent of signal inhibition and calculated using the standard curve.

### Sub-cellular fractionation

In order to quantify the amount of NTSRs expressed at the cell surface and intracellularly, we performed sub-cellular fractionation from brain homogenates. Plasma membranes were prepared from brain homogenates of WT or KO-NTSR3/Sortilin mice according to the protocol previously described (Clancy and Czech, 1990). 30 μg of crude homogenates, purified plasma membranes and high and low density vesicles (H/LDM) were submitted to Western blot analysis using the rabbit polyclonal antibodies against NTSR1 or NTSR2 (1:500) (SantaCruz Technologies (USA)). Proteins detected with these antibodies were normalized using antibodies specific for each intracellular compartment (NaKATPase for plasma membranes, TGN38 for H/LDM and tubulin for total extracts) from SantaCruz technologies (USA).

### Statistics

Results are expressed as mean ± standard error mean (SEM). Statistical analyses were performed using GraphPad (version 6.0). Student *t*-tests were used when appropriate to evaluate differences in quantitative variables whereas analysis of variance (ANOVA) was used to compute possible differences between groups.

## Results

### Binding of NT to brain homogenates from wild type and NTSR3/sortilin KO mice

**Figure 1:**
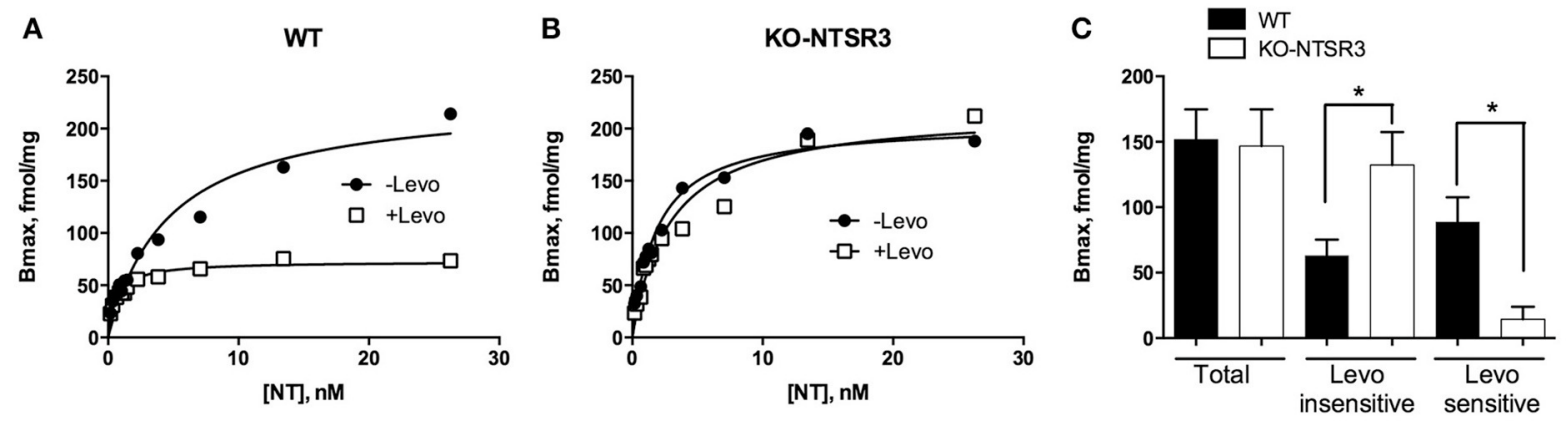
**Binding of ^125^I-NT to brain homogenates from WT and NTSR3/sortilin KO mice. (A,B)** Brain homogenates from WT **(A)** or from NTSR3/sortilin KO mice **(B)** (60 μg of proteins) were incubated with increasing concentrations of ^125^I-NT alone or isotopically diluted with unlabeled NT in the absence (closed symbols) or in the presence (open symbols) of 1 μM levocabastine. Saturation curves were made from specific binding using GraphPad analysis. **(C)** Representation of the mean ± SEM of total binding and levocabastine-sensitive and -insensitive binding sites calculated from 5 independent experiments performed in triplicate. ^*^p < 0.05 using Student *t*-Test.

In order to quantify the amount of NT binding sites corresponding to NTSR1 and NTSR2 in the brain of wild type (WT) and NTSR3/sortilin deficient mice (KO-NTSR3), we first performed saturation binding experiments of iodinated NT on homogenates prepared from the indicated brains in the absence or in the presence of the NTSR2 selective blocker levocabastine (1 μM) (Kitabgi et al., 1987). In brain homogenates from WT mice, in the absence of levocabastine, the saturation curve obtained from a typical experiment indicated a maximal binding capacity (Bmax) of about 200 fmol/mg (Figure 1A). In the presence of levocabastine, the Bmax decreased to 65–70 fmol/mg (Figure 1A), a binding capacity corresponding to the levocabastine insensitive NT binding sites attributed to NTSR1. Interestingly, in brain homogenates from KO-NTSR3 mice, saturation experiments performed in the absence or in the presence of levocabastine revealed the same Bmax (Figure 1B), demonstrating that in KO-NTSR3 mice, the binding of NT is insensitive to the drug. Figure 1C which summarized the Bmax mean values obtained from 5 independent experiments, clearly indicated that the amount of levocabastine-insensitive NT binding sites increased in KO mice (from 63 ± 12 fmol/mg in WT mice to 124 ± 30 fmol/mg in KO mice, *p* = 0.029). As expected, the amount of levocabastine-sensitive NT binding sites was decreased in brain from KO mice from 88 ± 19 fmol/mg in WT to 14 ± 9 fmol/mg in KO (*p* = 0.028).

### Measurement of the expression of NTSR1, NTSR2 in brain homogenates from wild type and NTSR3/sortilin KO mice

Binding experiments performed above suggested a loss of levocabastine-sensitive NT binding sites (i.e., NTSR2) and an increase of levocabastine-insensitive NT binding sites (i.e., NTSR1) in the brain of KO-NTSR3 mice. For this reason, we further analyzed the expression of both receptors at the mRNA and protein levels.

**Figure 2:**
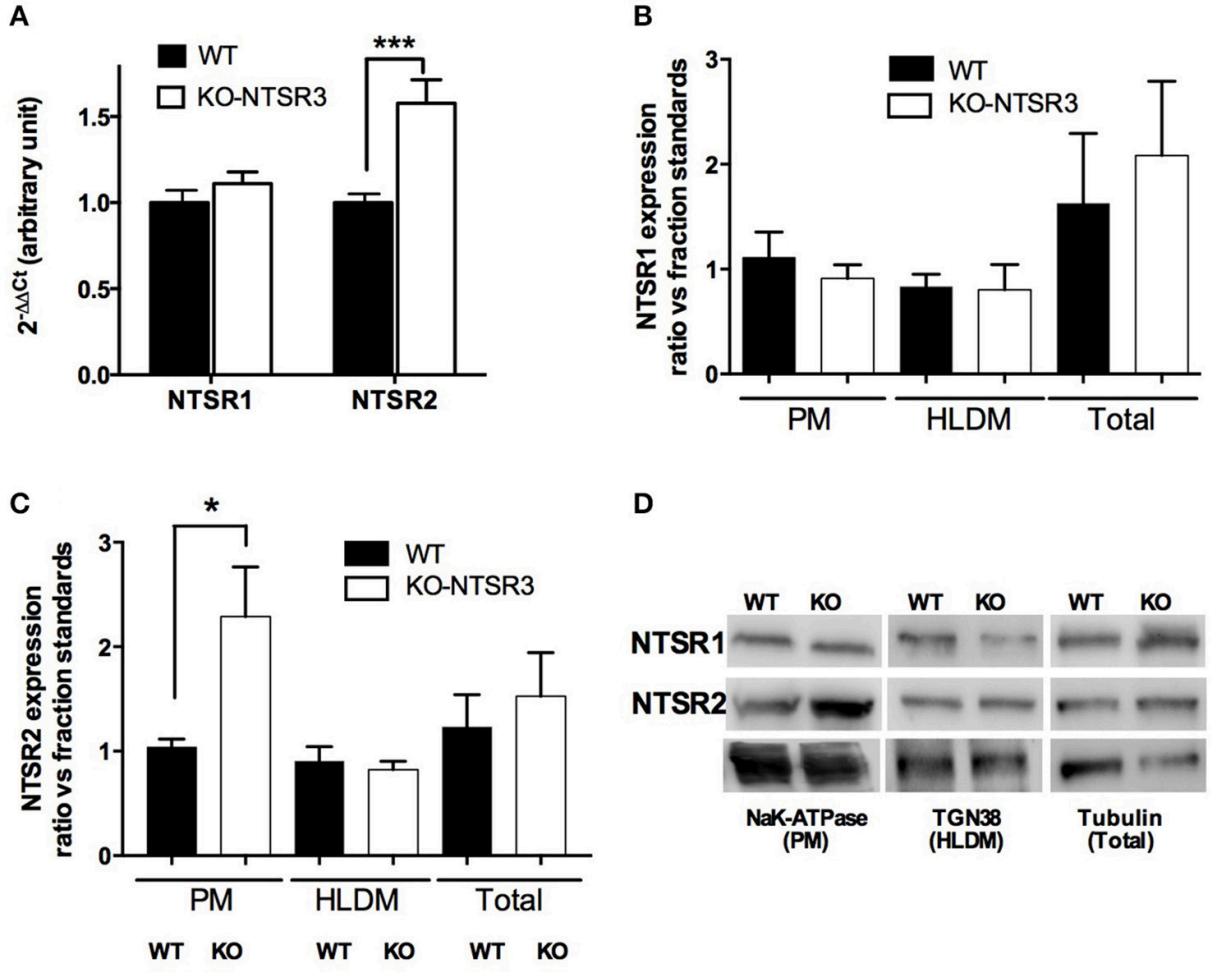
**Quantification of NT receptors from WT and NTSR3/sortilin KO mice (A)** Quantitative PCR of NTSR1 and NTSR2 from WT and NTSR3/sortilin KO mouse brains, ^***^p < 0.001. **(B,C)** Protein expression of NTSR1-like and NTSR2-like in plasma membranes (PM), high and low density vesicles (HLDM) and total homogenates prepared from brains from WT and NTSR3/sortilin KO mice. Each bar in the graphs represents the mean ± SEM of bands intensitiy quantified using the corresponding compartment markers from 5 independent experiments. ^*^p < 0.05 using Mann and Whitney Student *t*-test. **(D)** Representative Western blot analysis of NTSR1-like and NTSR2-like proteins expressed in plasma membranes (PM), high and low density vesicles (HLDM) and total homogenates prepared from brains from WT and NTSR3/sortilin KO mice. NaK-ATPase; Sodium Potassium-ATPase, TGN38; Trans-Golgi Network protein of 38 kDa.

Intriguingly, quantitative PCR (qPCR) determination indicated that the mRNA of NTSR1 remained unchanged whereas the amount of NTSR2 mRNA was significantly increased in the brain of KO mice (*p* < 0.001) (Figure 2A). This increase of NTSR2 mRNA was in contradiction with the loss levocabastine-sensitive NT binding sites. The similar mRNA level of NTSR1 between WT and KO mice did not correspond to the increase of levocabastine-sensitive NT binding sites observed in the brain of KO mice. Therefore, we verified the protein expression of both receptors after subcellular fractionation and Western blot analysis. The quantification determined from 5 independent experiments indicated that the protein level of NTSR1-like remained similar at the plasma membranes (PM), in the high and low density vesicles (H/LDM) and in the total extracts from brain from WT and KO mice (Figure 2B). However, the amount of NTSR2-like protein was significantly increased by a factor 2 (*p* < 0.05) at the plasma membranes prepared from KO mouse brain but was similar between WT and KO mice in H/LDM and total extracts (Figures 2C,D).

### Increased expression of NT in brain and serum from NTSR3/sortilin KO mice

**Figure 3:**
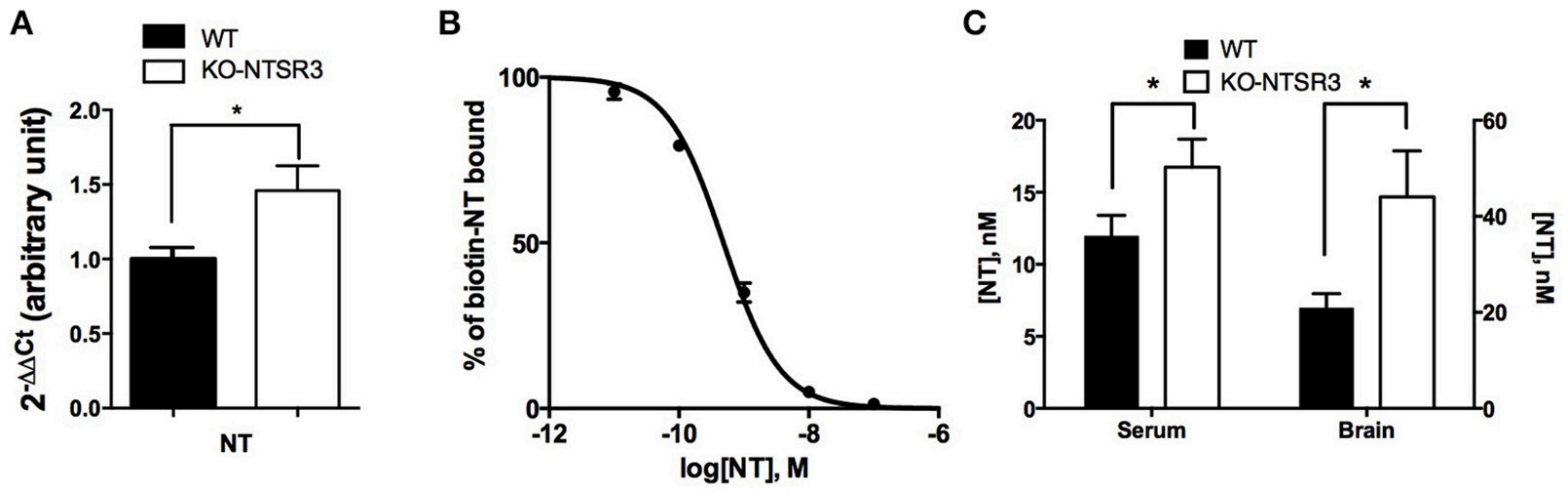
**Measurement of brain and blood NT content in WT and NTSR3/sortilin KO mice (A)** Quantitative PCR of NT from WT and NTSR3/sortilin KO mouse brains. **(B)** Competitive inhibition of biotinylated NT by unlabeled NT, the standard curve was the mean ± SEM from 3 independent experiments performed in triplicate, the corresponding IC50 was 0.48 nM. **(C)** NT concentrations in sera and in brain from WT and NTSR3/sortilin KO mice measured using AlphaLisa™ technique. Each bar in the graphs represents the mean value ± SEM of NT concentrations determination in serum (*n* = 19) and in brain extracts (*n* = 3). ^*^p < 0.05.

As we observed an important increase of NTSR2 expression at the plasma membrane, we wondered whether the expression of its ligand may also be modified in NTSR3/sortilin KO mice by using the dosing method developed for NT. We first observed that the amount of NT mRNA was also significantly enhanced in the brain of KO mice (*p* < 0.05) (Figure 3A). The higher level of NT mRNA measured in the brain from NTSR3/sortilin KO mice prompted us to quantify the peptide content in brain extracts and serum from both mice. To perform these experiments, we developed tools (specific antibodies and biotinylated NT) to be used according to the AlphaLisa™ method (Perkin). The Figure 3B illustrated the competition curve between biotinylated NT and unlabeled NT. The amount of NT present in the serum or in the brain extracts was determined from this curve (Figure 3C). We observed a significant increase of the peptide in serum (from 12 nM in WT to 18 nM in KO mice) and brain extracts (from 21 nM in WT to 45 nM in KO) (Figure 3C).

### NTSR3/sortilin KO mice are resistant to pain

**Figure 4:**
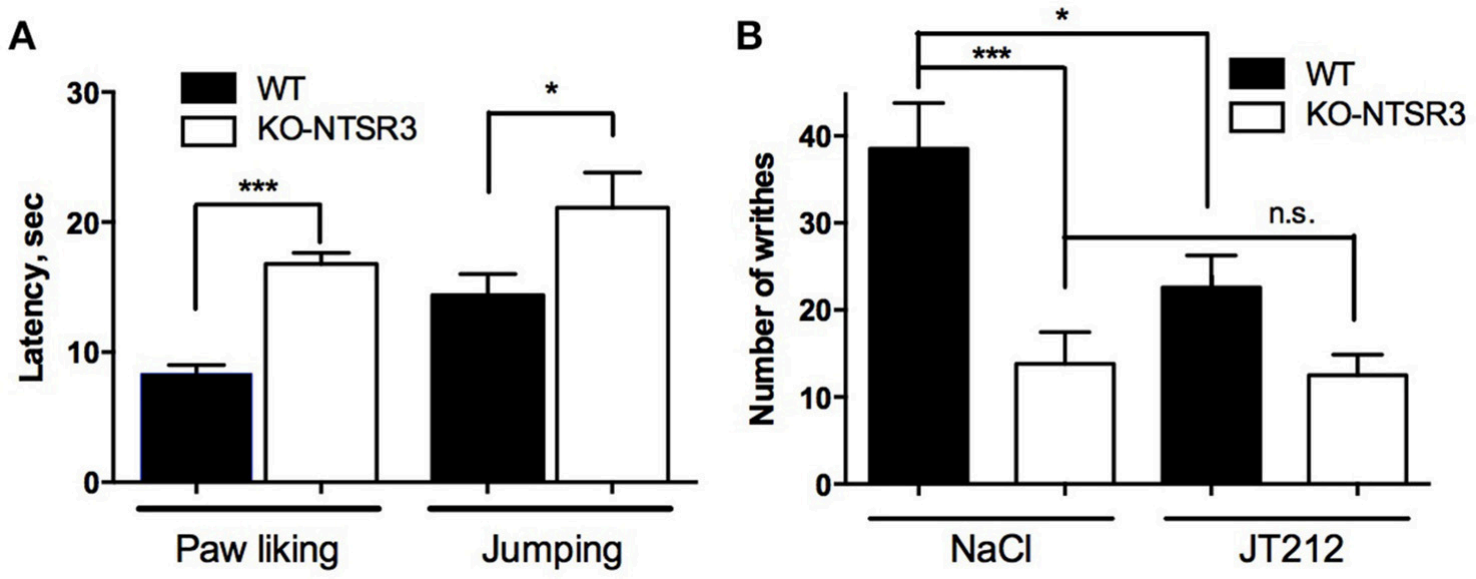
**Analgesic responses of WT and NTSR3/sortilin KO mice (A)** Hot plate test, mice were placed on a plate at a temperature of 55°C. Bars represent mean ± SEM of paw licking and jumping latencies. Paw licking latency, ^***^p < 0.001, *n* = 20; jumping latency, ^*^p < 0.05, *n* = 20. **(B)** Writhes were counted over a 15 min period after intraperitoneal injection of 0.5% acetic acid after intraperitoneal injection of either vehicle (NaCl) or 100 μl of 1 μM JT212. The number of indicated writhes is the mean ± SEM from groups of 10–12 mice. ^***^p < 0.001, ^*^p < 0.05, n.s: non-significant.

Since we observed an increase of both NT and NTSR2 in NTSR3/sortilin KO mice and that NTSR2 is mainly involved in NT-induced analgesia (Dubuc et al., 1999), we wondered whether NTSR2 is still functional using acute pain tests including chemical (writhing test) and thermal (paw licking) nociceptive tests. When mice were placed on the hot plate, the latency for paw licking increased from 8.9 ± 0.85 s for WT mice to 17.3 ± 0.95 s for KO mice (*p* < 0.001) (Figure 4A). Similarly, the latency to jump was 30.9 ± 1.5 s for WT mice and increased to 39.4 ± 3.7 s for KO mice (*p* = 0.038) (Figure 4A), suggesting a resistance to pain for KO-NTSR3 mice. When WT mice were subjected to the writhing test, the number of writhes/15 min was 38.5 ± 5.2 (Figure 4B). In KO-NTSR3 mice, the number of writhes was 13.8 ± 3.6, a value significantly different to that obtained in WT mice (*p* < 0.001). Therefore, we tested the effect of IP injection of JT212 (100 μl of a 1 μM solution) on the pain writhing test and as expected, JT212 significantly decreased the number of writhes to 22.6 ± 3.7 in WT mice (One way ANOVA, *p* = 0.028) (Figure 4B). In KO mice, the injection of the peptide was without significant effect on the number of writhes (12.5 ± 2.4 (*p* = 0.99) (Figure 4B) indicating that no further analgesic action of JT212 was measurable when animals were already desensitized.

## Discussion

In the present work, we provide evidence that the absence of NTSR3/sortilin leads to modificiation of the neurotensinergic system with the consequence that these mice are less sensitive to pain as clearly shown by the two different tests (Figure 4). This particular behavior is likely due to the increase of both NTSR2, the NT receptor mainly involved in the analgesic effect of NT (Dubuc et al., 1999), and NT itself.

However, in the first series of experiments we performed to analyze the amount of levocabastine-sensitive and -insensitive NT binding sites from WT and KO mice brains, we obtained contradictory results. Binding experiments revealed that in the brain from KO mice, the amount of levocabastine-sensitive binding sites, predicted to be NTSR2 (Kitabgi et al., 1987; Mazella et al., 1996), was dramatically decreased whereas levocabastine-insensitive binding sites appeared to be enhanced (Figure 1). By constrast, qPCR and Western blot analyses indicated no change in the NTSR1 content and a significant increase of NTSR2 at the plasma membranes prepared from NTSR3/sortilin KO mouse brain (Figure 2). A possible explanation is that the sensitivity of NTSR2 to levocabastine as well as its relatively low affinity to NT are likely due to its interaction with NTSR3/sortilin as already observed in beta cells (Beraud-Dufour et al., 2009). In the absence of NTSR3/sortilin, NTSR2 could be less retained intracellularly and the conformation of NTSR2 protein could prevent the binding of levocabastine and could increase its affinity for NT. Growing evidences have demonstrated that homo and heterodimerizations of GPCRs are involved in receptor recognition, cellular trafficking and signaling (for review see Fuxe et al., 2014). Concerning the neurotensinergic system, NTSR1 has been shown to be functionally associated with dopamine D2 receptor to modulate its activity (Borroto-Escuela et al., 2013). Heterodimerization between NTSR1 and NTSR2 was also observed leading to modifications of intracellular NTSR1 distribution, trafficking and functionality (Perron et al., 2007; Hwang et al., 2010). In the present case, the increase of NTSR2 expression could lead to a general dysfunction of the neurotensinergic system by decreasing also the activity of NTSR1. A similar interaction between NTSR3/sortilin and NT receptors has been already demonstrated for NTSR1 expressed in the colonic adenocarcinoma cell line HT29 in which its physical association with NTSR3/sortilin led to a decrease of both the affinity of NT for NTSR1 and the NTSR1-mediated biological response (i.e., IPs turnover) (Martin et al., 2002).

Another interesting finding was the increase in the expression of NTSR2 concomittant to an increase of NT content both in the brain and in the blood from NTSR3/sortilin KO mice (Figure 3). The higher level of NT in the brain was correlated with the higher mRNA content for the peptide whereas the origin of the higher amount of NT measured in the serum from NTSR3/sortilin KO mice remains to be elucidated.

From the latter observations, we hypothesized that NTSR3/sortilin KO mice would likely behave differently than WT mice when subjected to pain, with modified sensitivity to thermal and chemical stimuli. As expected, there is a lower sensitivity of KO mice vs. WT mice to high temperatures as measured by paw licking and jump latency, and to intraperitoneal injection of acetic acid as measured by the number of writhes, corresponding to expected results from mice with a high content of NT (Kleczkowska and Lipkowski, 2013). These results confirm the importance of the neurotensinergic system in the control of pain modulation. The involvement of both NTSR1 and NTSR2 in the effect of NT on analgesia has been largely demonstrated in the literature either by using ligands selective for each receptor (Sarret et al., 2005; Smith et al., 2012) or by using mice in which NTSR1 or NTSR2 genes have been deleted (Maeno et al., 2004; Roussy et al., 2010).

In conclusion, the work presented here incorporated a new physiological concept that should be taken into account for further investigations for the development of NT analogs to be used in pain treatment. This concept is that a small increase of NT production in the brain, associated with an increase of NTSR2 expression, appears to be sufficient to reduce the sensitivity of animals to pain.

## Ethics statement

The local Ethics Committee (CIEPAL) (protocol number 00893.02).

## Author contributions

CD and JM designed study concept and supervised acquisition of the results. Acquisition of data by CD, SM, MR, and ED. JM wrote the manuscript with the help of CD, TD, and CM.

### Conflict of interest statement

The authors declare that the research was conducted in the absence of any commercial or financial relationships that could be construed as a potential conflict of interest.
